# Questioning the sex-specific differences in the association of smoking on the survival rate of hospitalized COVID-19 patients

**DOI:** 10.1371/journal.pone.0255692

**Published:** 2021-08-05

**Authors:** Athar Khalil, Radhika Dhingra, Jida Al-Mulki, Mahmoud Hassoun, Neil Alexis

**Affiliations:** 1 Clinical Research Unit, Rafik Hariri University Hospital, Beirut, Lebanon; 2 Department of Environmental Sciences and Engineering, Gillings School of Public Health, University of North Carolina, Chapel Hill, North Carolina, United States of America; 3 Institute for Environmental Health Solutions, University of North Carolina, Chapel Hill, North Carolina, United States of America; 4 Department of Pulmonary and Intensive Care Unit, Rafik Hariri University Hospital, Beirut, Lebanon; 5 Center for Environmental Medicine Asthma and Lung Biology, University of North Carolina, Chapel Hill, North Carolina, United States of America; National Yang-Ming University, TAIWAN

## Abstract

**Introduction:**

In the absence of a universally accepted association between smoking and COVID-19 health outcomes, we investigated this relationship in a representative cohort from one of the world’s highest tobacco consuming regions. This is the first report from the Middle East and North Africa that tackles specifically the association of smoking and COVID-19 mortality while demonstrating a novel sex-discrepancy in the survival rates among patients.

**Methods:**

Clinical data for 743 hospitalized COVID-19 patients was retrospectively collected from the leading centre for COVID-19 testing and treatment in Lebanon. Logistic regression, Kaplan-Meier survival curves and Cox proportional hazards model adjusted for age and stratified by sex were used to assess the association between the current cigarette smoking status of patients and COVID-19 outcomes.

**Results:**

In addition to the high smoking prevalence among our hospitalized COVID-19 patients (42.3%), enrolled smokers tended to have higher reported ICU admissions (28.3% vs 16.6%, p<0.001), longer length of stay in the hospital (12.0 ± 7.8 vs 10.8 days, p<0.001) and higher death incidences as compared to non-smokers (60.5% vs 39.5%, p<0.001). Smokers had an elevated odds ratio for death (OR = 2.3, p<0.001) and for ICU admission (OR = 2.0, p<0.001) which remained significant in a multivariate regression model. Once adjusted for age and stratified by sex, our data revealed that current smoking status reduces survival rate in male patients ([HR] = 1.9 [95% (CI), 1.029–3.616]; p = 0.041) but it does not affect survival outcomes among hospitalized female patients([HR] = 0.79 [95% CI = 0.374–1.689]; p = 0.551).

**Conclusion:**

A high smoking prevalence was detected in our hospitalized COVID-19 cohort combined with worse prognosis and higher mortality rate in smoking patients. Our study was the first to highlight potential sex-specific consequences for smoking on COVID-19 outcomes that might further explain the higher vulnerability to death from this disease among men.

## Introduction

Until we reach a global herd immunity or until an effective treatment for coronavirus disease (COVID-19) has been identified and proven efficacious, identifying and managing clinical risk factors as a supportive treatment strategy can help reduce the mortality rate among those severely affected by COVID-19 [1]. Smoking, as an avoidable risk factor, was proposed to be targeted as an effective intervention strategy that can impact poor outcomes for those with COVID-19 [2, 3]. Initially, some clinical and epidemiological studies claimed that smokers were less likely to be hospitalized due to COVID-19 while others refuted these findings by reporting a high smoking prevalence in their hospitalized COVID-19 cohorts compared to that of the study’s general population [4, 5]. In terms of prognosis and mortality, contradictory results were also reported. For example, Lippi et al. pointed out that active smoking is not associated with severity of the disease while others showed that smoking patients might have higher risk for mortality compared to non-smokers [3, 6]. These research studies, meta-analysis and systematic reviews emphasized on the importance of evaluating larger COVID-19 cohorts among different populations, especially from low socioeconomic countries where smoking presents a major public health burden [7–9].

The high smoking prevalence in the Lebanese population combined with the low socioeconomic status of this country encouraged us to study the effect of smoking on COVID-19 outcomes in the resultant cohort [10, 11]. Thus, we investigated the impact of smoking on 743 hospitalized COVID-19 patients from Lebanon with respect to their disease outcomes and mortality rates. On the other hand, sex and smoking have been independently proposed as risk factors for increased susceptibility and host severity to SARS-CoV-2 infection, yet none of these studies has interrogated the interaction between these two factors on COVID-19 outcomes [2]. Knowing that smoking adverse health consequences vary significantly between men and women, we investigated if this discrepancy plays a role when it comes to COVID-19 mortality rates. By examining a large representative cohort from the Middle-East region, we were the first to highlight a potential novel sex-discrepancy in the association of smoking on the survival rate of hospitalized COVID-19 patients and to confirm that smoking might be a critical risk factor for COVID-19 mortality.

## Methods

### Study participants

In this pilot study we collected retrospectively data for all hospitalized COVID-19 adults (defined as > 15 years old) who were admitted to Rafik Hariri University Hospital (RHUH). RHUH is the main governmental public hospital in Lebanon that was assigned as the leading center for COVID-19 testing and treatment in the country [12].

Inclusion Criteria: A diagnosis for COVID-19 was established when a patient tested positive for SARS-CoV-2 by PCR analysis of nasopharyngeal swab and had COVID-19 pneumonia according to the guidelines issued by the National Health Commission of the People’s Republic of China (PRC). Our study patients were those classified as being moderate, severe or critical COVID-19 cases as per the interim guidelines of the World Health Organization and the National Health Commission of China [13, 14]. Briefly, 1) moderate cases were patients with fever, respiratory symptoms, and imaging presentations of pneumonia that needed or were at high risk for oxygenation, 2) severe cases were those with any of the following clinical presentations: respiratory distress with RR>30 time/min, oxygen saturation at rest <93%, or PaO2/FiO2 <300 mmHg(I mmHg = 0.133 kPa), and 3) critically severe cases were patients with any of the following: respiratory failure needing mechanical ventilation, shock, or combination with other organ failure. Overall, both severe and critical cases were admitted to the Intensive Care Unit (ICU). Mild cases were excluded from this study since the investigated outcomes (ICU admission and mortality) are not usually reported among these patients. All the enrolled patients received supportive and therapeutic modalities based on an inpatient guide and a defined treatment strategy for all patients admitted to the hospital at the same period of time.

### Data

The electronic medical records of COVID-19 patients admitted to RHUH between the 1st of February until the 31 of October 2020 were accessed in November 2020. The data was de-identified by the study coordinator before the authors have had access to it. The obtained data included patient demographics, self-reported smoking history (current cigarette smoking), ICU admission, total number of days spent in the hospital, and the final outcome was defined as death or discharged. Outcomes for current smokers were compared to that of never and former smokers combined together. The study was conducted according to the principles outlined in the Declaration of Helsinki, and was approved by the Rafik Hariri University Hospital Institutional Review Board.

Patients who were hospitalized for isolation and quarantine reasons were excluded from the study. Seven hundred forty-three (N = 743) patients met the study inclusion criteria and were analyzed for study outcomes. No missing data were reported among all enrolled patients.

### Statistical analysis

Categorical variables were presented as absolute numbers or percentages. Continuous variables were expressed as mean values ± Standard Deviation (SD). Differences of the studied parameters between discharged and dead COVID-19 patients were evaluated by student’s t-test for parametric data. Logistic regression was used for each studied parameter over the binary outcome (discharged/death) using a univariate analysis. Multivariable logistic regression analyses were used to assess the association between age, sex, smoking and the need for ICU admission or the reported death incidence. Odds Ratios (OR) with 95% Confidence Intervals (CI) were reported. Survival curves were visually presented by Kaplan-Meier curves. The survival distributions of smokers and non-smokers were compared using the Mantel-Cox log-rank test. Survival analysis was conducted using Cox proportional hazards models that were adjusted for age and stratified by sex. Assessing interaction between smoking and sex was conducted in an additional age-adjusted Cox proportional hazards model that included the sex factor. Hazard ratio (HR) and the 95% CI were reported. All tests were two tailed and considered significant when p-value <0.05. All data were analyzed using SPSS software (Version 23.0, IBM).

## Results

### General characteristics of hospitalized COVID-19 patients

Seven hundred forty-three patients met the study inclusion criteria. The mean age of all enrolled patients was 49.7 ± 19.04 years with the majority of 463 patients (62.3%) being men. The mean number of days stayed in the hospital among our patients was 11.32± 8.17 days while only 160 (21.5%) patients needed admission to the ICU. Death was reported among 81 patients (17.4%). Of all patients, 314 (42.3%) reported to be current active cigarette smokers. There was no difference in mortality rate between males and females while the mean age of patients who died was higher than those who survived (65.4 ± 15.77 vs 47.8 ± 18.53 years, p<0.001). Compared to discharged patients, those who died presented a higher percentage of smoking behavior (60.5 vs 40%, p<0.001) and a longer length of stay in the hospital (14.98 ± 10.52 vs 10.87 ±7.73 days) with more frequent need for ICU admission (80.2% vs 14.4%, p<0.001) (Table 1).

**Table 1:** Characteristics of the hospitalized COVID-19 patients (n = 743) according to their final outcome, smoking status, and sex. SD = standard deviation.

			Final outcome			Smoking Status			Sex		
Variables		All patients (100%)	Discharged n = 662	Deceased n = 81	P value	Non-smoker n = 429	Smoker n = 314	P value	Male n = 463	Female n = 280	P value
**Sex**											
	**Male, n (%)**	463 (62.3%)	411 (62.1%)	52 (64.2%)	0.711	241 (56.21%)	222 (70.7%)	<0.001	463 (100%)	0 (0%)	-
	**Female, n (%)**	280 (37.7%)	251 (37.9%)	29 (35.8%)		188 (43.8%)	92 (29.3%)		0 (0%)	280 (100%)	
**ICU admission**											
	**No, n (%)**	583 (78.5%)	567 (85.6%)	15 (19.8)	<0.001	358 (83.4%)	225 (71.7%)	<0.001	97 (21%)	63 (22.5%)	0.618
	**Yes, n (%)**	160 (21.5%)	95 (14.4%)	65 (80.2%)		71 (16.6%)	89 (28.3%)		366 (79.0%)	217 (77.5%)	
**Final outcome**											
	**Discharged, n (%)**	662 (89.1%)	662 (100%)	0 (0%)	-	397 (92.5%)	265 (84.4%)	<0.001	411 (88.8%)	251 (89.6%)	
	**Deceased, n (%)**	81 (10.9%)	0 (0%)	81 (100%)		32 (7.5%)	49 (15.6%)		52 (11.2%)	29 (10.4%)	0.711
**Smoking status**											
	**Non-smoker, n (%)**	429 (57.7%)	397 (60%)	32 (39.5%)	<0.001	429 (100%)	0 (0%)	-	241(52.1%)	188 (67.1%)	0.001
	**Smoker, n (%)**	314 (42.3%)	265 (40%)	49 (60.5%)		0 (0%)	314 (100%)		222 (47.9%)	92 (32.9%)	
**Age, mean** ± **SD**		49.7 ± 19.0	47.8 ± 18.5	65.4 ± 15.8	<0.001	46.3 ± 19.1	54.3 ± 18.0	<0.001	49.4 ± 17.9	50.2 ± 20.8	0.551
**Number of days in hospital, mean** ± **SD**		11.3 ± 8.2	10.9 ± 7.7	15.0 ± 10.5	0.001	10.8 ± 8.4	12.0 ± 7.8	0.041	10.4 ± 8.5	8.2 ± 6.8	0.066

### Characteristics of hospitalized COVID-19 patients as per their smoking status

**Fig 1:**
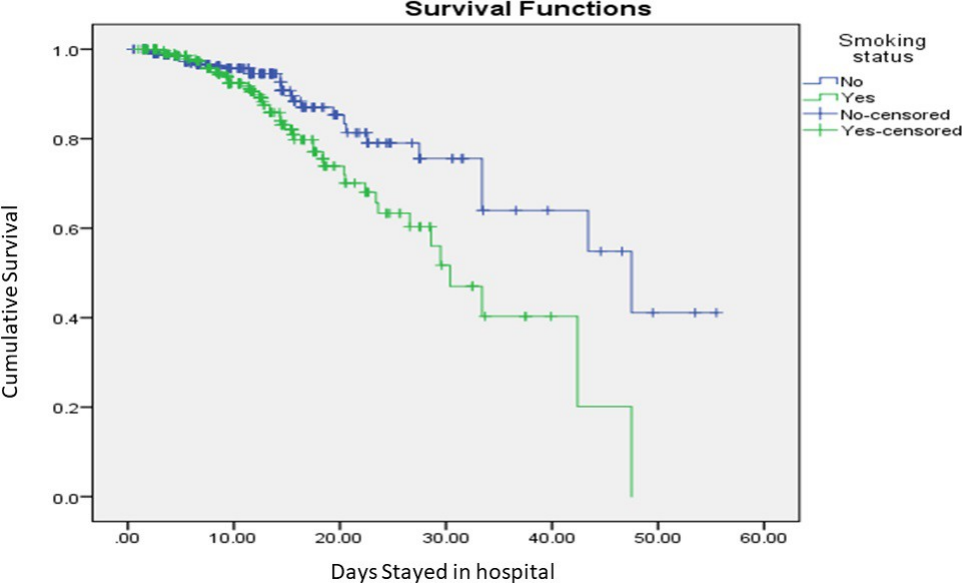
Kaplan-Meier survival curve for hospitalized smokers (green line) and non-smokers (blue line). p = 0.004.

Smokers were more likely to be males (70.7% males vs 29.3% females) and to have a higher mean age (54.31 ±17.98 years) as compared to nonsmokers (46.30 ±19.12 years). Death incidence among smokers was approximately double that of non-smokers (15.6% vs 7.5%). ICU admission was higher among smokers (28.3%) with a longer length of stay in the hospital (12.03 days ±7.78) (Table 1). In a univariate logistic regression analysis, the odds ratio of death (OR = 2.3, 95% CI = 1.43–3.68, p<0.001) and ICU admission (OR = 2, 95% CI = 1.400–2.841, p<0.001) was high among smoker as compared to non-smokers (Table 2). In a multivariate analysis, age and smoking status remained significantly associated with death and ICU admission. The sex factor by itself or in combination with the other factors was not associated with the detected outcomes. To compare the survival times between smokers and non-smokers, Kaplan-Meier estimates were plotted (Fig 1). A worse survival rate was observed among smokers with a median survival of 30.4 days (95% CI 25.4 to 35.4 days) as compared to 47.5 days (95% CI 29.8 to 65.2 days) in non-smokers. The significance of survival trends that was observed in between the groups was confirmed by Mantel-Cox log-rank test (p = 0.004).

**Table 2:** Univariate and multivariate logistic regression demonstrating odds ratio (OR) for death and ICU admission among hospitalized COVID-19 patients (n = 743). OR = odd ratio.

Variables	ICU Admission						Death					
	Univariate logistic regression			Multivariate logistic regression			Univariate logistic regression			Multivariate logistic regression		
	OR	95% confidence intervals	P-Value	OR	95% confidence intervals	P-Value	OR	95% confidence intervals	P-Value	OR	95% confidence intervals	P-Value
**Age**	1.044	1.033–1.055	<0.001	1.042	1.031–1.053	<0.001	1.05	1.039–1.068	<0.001	1.053	1.038–1.068	<0.001
**Male sex**	1.095	0.765–1.568	0.619	0.917	0.622–1.353	0.664	1.095	0.677–1.771	0.711	1.172	0.697–1.965	0.549
**Smoking**	1.994	1.400–2.841	<0.001	1.584	1.084–2.314	0.017	2.294	1.431–3.677	<0.001	1.7	1.029–2.808	0.038

### The influence of Sex on COVID-19 mortality rate with respect to the smoking status

**Fig 2:**
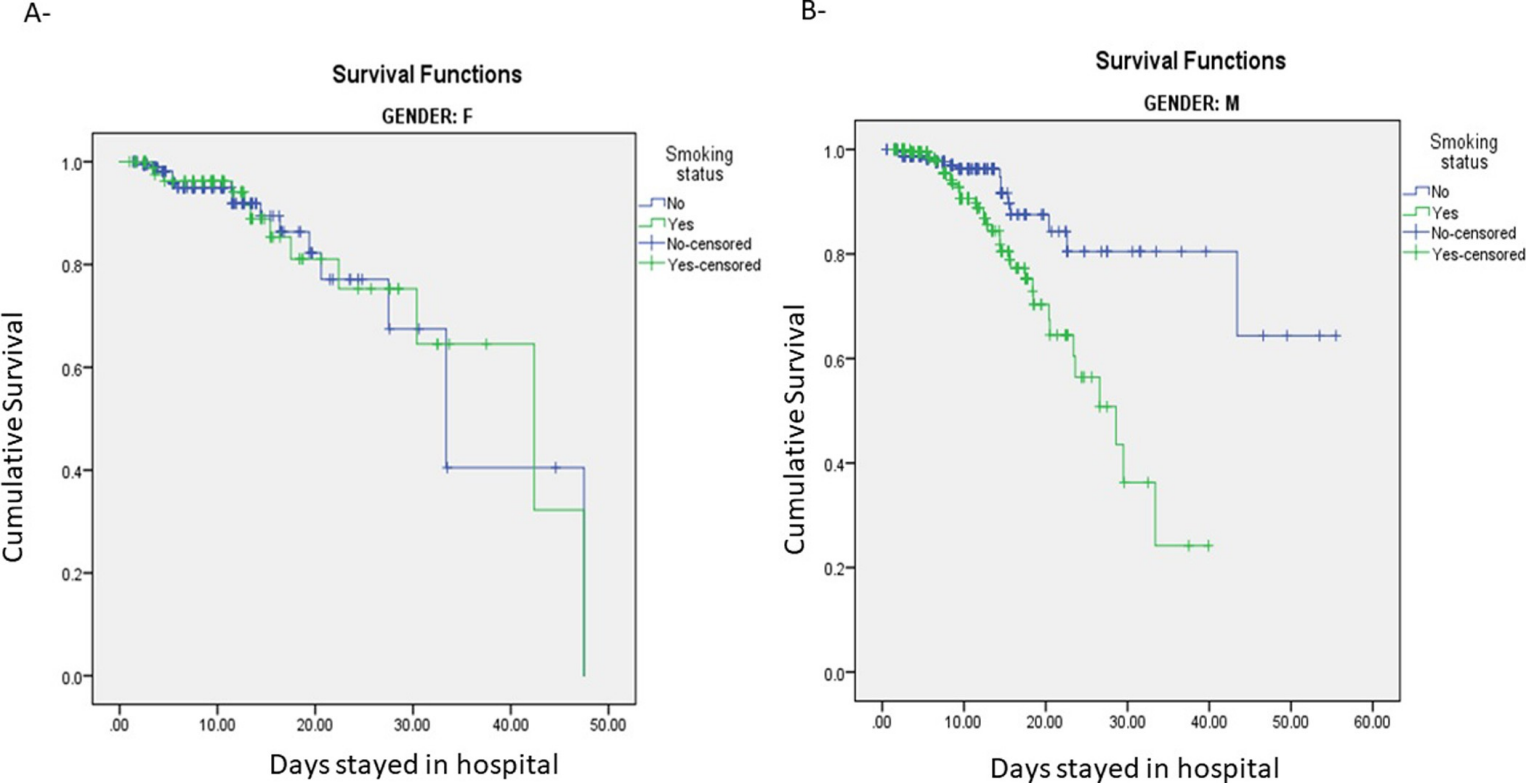
Kaplan-Meier survival curve for smokers (green line) and non-smokers (blue line) as stratified by Sex. A) F = Female (p = 0.893). B) M = Male (p = 0.001).

Smoking adverse health consequences are known to vary significantly between men and women [15]. Thus, we investigated whether this discrepancy plays a role when it comes to COVID-19 mortality rates. Our cohort presented a comparable profile between males and females with no significant difference in age, ICU admission, length of stay in hospital and death incidence (Table 1). Once stratified by sex, only the survival analysis among men showed a significant difference between smokers and non-smokers (Fig 2). In a univariate logistic regression analysis, the odds ratio for death (OR: 3, 95% CI = 1.6–5.7, p = 0.001) and ICU admission (OR: 2.3, 95% CI = 1.4–3.6, p = 0.001) was significantly higher in smoking men as compared to non-smoking men (S1 Table). Multivariable Cox proportional hazard regression model adjusted for age, the most significant risk factor for mortality among COVID-19 patients, demonstrated that current smoking is an independent risk factor for reduced survival time from admission to death in men (hazard ratio [HR], 1.9 [95% confidence interval (CI), 1.029–3.616]; P = 0.041). The Cox model evaluating the interaction of sex and smoking status was also significant (p = 0.015). Neither the logistic regression analysis nor the Cox proportional hazard regression model adjusted for age showed a significant difference in the survival rate among women with respect to their smoking status ([HR], 0.79 [95% CI = 0.374–1.689]; p = 0.551) (S1 Table).

## Discussion

Lebanon as a developing country with a low-middle income profile was ranked third globally for having the most smokers per capita for both sexes. This was declared after the striking increase of 475% in cigarette consumption in this country during the last few decades [16]. The smoking prevalence among the Lebanese women ranked as the highest among the Middle Eastern countries and exceeded that of women residing in the United States [10, 11]. Thus, evaluating the association between smoking and COVID-19 outcomes in this country is highly representative and informative [16]. In this study, the smoking prevalence (42.3%) among 743 hospitalized COVID-19 patients exceeded the prevalence of tobacco users (38%) in the Lebanese general population and was higher than those reported by other COVID-19 cohorts [17, 18] Additionally, elevated odds ratios for ICU admission and death was detected among our smoking patients by both univariate and multivariate analysis. Thus, our results were consistent with most of the well-designed epidemiological studies that revealed smoking as an independent risk factor that can worsen in-hospital outcomes and increases mortality rates among COVID-19 patients [3, 19–22]. Collectively, such results infer adding smoking as a risk factor for worse COVID-19 prognosis and highlight the importance of smoking cessation amidst this pandemic [22]. Yet, none of these studies investigated the results consistency between smoking men and women. In this study we were the first to report a potential noteworthy sex-discrepancy in the association of smoking on COVID-19 mortality rates. Interestingly, current smoking status was not associated with the need for ICU admission or with the survival outcomes among hospitalized COVID-19 female patients but rather showed that smoking men have reduced worse outcomes as compared to their non-smoking counterpart.

Smoking adverse health consequences vary frequently between men and women [15]. The widely accepted hypothesis reveals that although men have higher smoking prevalence compared to women, the latter are at higher risk for smoking-related general health problems [23]. However, concerning lung diseases, men may be more susceptible. For example, it has been proposed that maternal smoking during pregnancy has a greater tendency to cause lifelong illness among males rather than females [24]. Another study pinpointed that smoking men have more emphysematous deterioration of the lungs compared to smoking women [25]. A significant representative cohort of 22,708 individuals demonstrated that smoking women were more vulnerable for earlier death and risk of stroke as compared to smoking men, but less vulnerable for lung disorders [26]. Since COVID-19 targets mainly the lungs, smoking-related lung disorders overlap with COVID-19 respiratory comorbidities that include chronic bronchitis, emphysema and chronic obstructive pulmonary disease (COPD) [27, 28]. Additionally, a more recent study by Naidu et. showed that the induction of angiotensin converting enzyme 2 (ACE-2) in mouse lungs after e-cigarette vapor exposure is sex-specific. A more prominent nicotine-dependent increase in lung ACE-2 expression was detected in male mice as compared to female mice which can influences both SARS-CoV-2 infectivity and COVID-19 severity [29]. Thus, the identified salient consequences for smoking among men in our cohort could be explained by the fact that this group might have higher odds for developing lung disorders in response to tobacco smoking and/or due to the more prominent increase in the ACE-2 expression among smoking male patients.

The main limitation of this study is the retrospective collection of our data which prevented us from further examining the implication of smoking habits (mild/heavy consumption, current/former) and comorbidities on the detected results. Although we cannot ignore a potential cofounding effect for these variables, many cohorts that accounted for comorbidities in their analysis were still able to declare smoking as an independent risk factor that is not affected by specific characteristics of the patients [3, 30, 31]. Taking into consideration these newly emerging reports and our relatively large size cohort, we anticipate that our results will not be highly affected once adjusted for other comorbidities. While our ability to detect a difference in mortality by smoking status among females may have been limited by the number of hospital-admitted women in our stratified analyses, our pooled analysis demonstrated a significant interaction of smoking status with sex. Furthermore, our sex-stratified results are consistent with a recent study by Magfira et. al who demonstrated revealed a positive correlation between the prevalence of adult male smoking and COVID-19 lethality in lower-middle-income countries (LMIC) which was not detected among smoking females. This study showed that each percentage point increase in adult male smoking prevalence caused the Case fatality rate (CFR) of COVID-19 to increase by 0.08% (95% CI 0.00%-0.15%, p = 0.041) [32]. Taking into consideration that our study was done in a LMIC, our results were consistent with this comprehensive study to indicate a potential sex sensitivity for smoking in COVID-19 cases that is worth noting and investigating in prospective studies. On the other hand, tobacco dosage consumption might have a potential cofounding effect on COVID-19 morbidity and thus, accounting for smoking dosage might underlie the prominent morbidity among male COVID-19 patients since men tend to have higher rates of cigarettes consumption per day as compared to women [26]. Previous studies have showed that the detected global sex disparities in severity and mortality from COVID-19 could be driven by the higher presence of comorbidities (i.e. cardiovascular disease, hypertension, diabetes and chronic lung disease) in men or due to the high-risk behaviors including smoking and alcohol use among this group. Yet, data from a recent large cohort indicated that sex-disparity in COVID-19 remained significant even after controlling for these potential confounders. In their study, the authors encourage further investigations on the biological pathways that may influence disease etiology rather than pointing on the effect of other factors and comorbidities [33]. Thus, our data could further highlight the importance of such investigations, especially that sex-discrepancy in the effect of smoking on heath outcome is a widely studied and accepted phenomena.

Identifying patients who are at higher risk for COVID-19 mortality will enable clinicians to intervene, allocate resources, and make more informed triage decisions [34]. Revealing smoking as a modifiable risk factor for men can enhance our control to this disease by highlighting the importance of smoking cessation specifically among this group while categorizing priorities during hospital admission. Our data suggest that adding sex-specific consequences for smoking on COVID-19 outcomes could further explain why men are more vulnerable to death from this disease globally. This pilot study will open the door for further investigations on the potential sex-discrepancy in the effect of smoking in COVID-19 while accounting for comorbidities and smoking dosages.

## Supporting information

S1 Table Univariate logistic regression for ICU admission and death among COVID-19 patients as stratified by sex and the Cox proportional hazard regression model. OR = Odds ratio, HR = Hazard ratio.
(DOCX)
