# Supplementary material for: Questioning the sex-specific differences in the association of smoking on the survival rate of hospitalized COVID-19 patients
Source: PLoS One. 2021 Aug 5;16(8):e0255692. doi: 10.1371/journal.pone.0255692 (PMC8341532; doi:10.1371/journal.pone.0255692)
Supplement: S1 Table — OR = Odds ratio, HR = Hazard ratio. (DOCX) [file pone.0255692.s001.docx]

|  | **Smoking women (n=92)** | | | | | | **Smoking men (n=222)** | | | | | |
| --- | --- | --- | --- | --- | --- | --- | --- | --- | --- | --- | --- | --- |
|  | Univariate logistic regression | | | Cox proportional hazard regression | | | Univariate logistic regression | | | Cox proportional hazard regression | | |
|  | OR | 95% confidence intervals | P-Value | HR | 95% confidence intervals | P-Value | OR | 95% confidence intervals | P-Value | HR | 95% confidence intervals | P-Value |
| Death | 1.5 | 0.688-3.309 | 0.3 | 0.79 | 0.374-1.689 | 0.551 | 3 | 1.604-5.661 | 0.001 | 1.9 | 1.029-3.616 | 0.041 |
| ICU admission | 1.8 | 0.985-3.124 | 0.6 | - | - | - | 2.3 | 1.435-3.624 | <0.001 | - | - | - |
